# Evidence for Strand Asymmetry in Different Plastid Genomes

**DOI:** 10.3390/genes14020320

**Published:** 2023-01-26

**Authors:** Cindy Ruan, Brian R. Morton

**Affiliations:** Department of Biology, Barnard College, Columbia University, 3009 Broadway, New York, NY 10027, USA

**Keywords:** plastid genome, strand asymmetry, base composition, genome structure, genome evolution, DNA replication

## Abstract

A common genome composition pattern in eubacteria is an asymmetry between the leading and lagging strands resulting in opposite skew patterns in the two replichores that lie between the origin and terminus of replication. Although this pattern has been reported for a couple of isolated plastid genomes, it is not clear how widespread it is overall in this chromosome. Using a random walk approach, we examine plastid genomes outside of the land plants, which are excluded since they are known not to initiate replication at a single site, for such a pattern of asymmetry. Although it is not a common feature, we find that it is detectable in the plastid genome of species from several diverse lineages. The euglenozoa in particular show a strong skew pattern as do several rhodophytes. There is a weaker pattern in some chlorophytes but it is not apparent in other lineages. The ramifications of this for analyses of plastid evolution are discussed.

## 1. Introduction

Many bacterial genomes display a pattern of strand asymmetry that is an effect of their bidirectional replication from a single origin of replication. A genome with a single replication origin is divided into two halves, called replichores, with boundaries that are defined by the locations of the origin and the terminus of replication, which is found directly opposite the origin on the circular chromosome. In some species, the genome displays a ‘skew’ meaning that either or both of the parity rules that A = T and G = C on one strand of a double-stranded DNA molecule, does not hold true. Additionally, the skew is associated with the leading/lagging strand arrangement in the two replichores such that the leading strand of each replichore has the same skew [1,2], such as a ‘GT’-skew in which T > A and G > C, with the lagging strand, of course, displaying the complementary skew.

This skew pattern results from a combination of differences in mutation dynamics between the strands and/or selection [1,3] and is typically visualized using sliding window skew plots of the cumulative values of both AT skew ([A − T]/[A + T]) and GC skew ([G − C]/[G + C]) along the length of the genome. Genomes with the skew (or asymmetry) pattern described above display a V-shaped shape (or the inverse) across the genome length [1,3,4] since the skew on one strand flips at the origin and terminus. We refer here to the composition structure that yields a V-shaped plot as a skew structure. This pattern has also been used to identify a putative location of replication origin in bacteria [4,5].

This skew structure is sometimes accompanied by a coding strand bias, in which genes tend to be coded on either the leading or lagging strand, but the two features are not necessarily related [4]. In fact, the asymmetry is typically strongest in putatively neutral sites, such as intergenic DNA or third codon positions of coding regions, indicating that the asymmetry is not due solely to any feature of protein coding but that there is a difference in mutation properties between the leading and lagging strands [1,4,6].

The plastid genome arose via a single primary endosymbiotic event involving a cyanobacterial-like prokaryote [7] and, although it is typically greatly reduced in size relative to bacterial chromosomes, it still retains many ancestral characteristics. Most interesting for this study is that the genome has a circular structure in most cases, the main exception being diatoms which appear to have an unusual and complex arrangement of linear and circular molecules [8]. The angiosperm chloroplast genome is replicated by a relatively complex D-loop mechanism [9] instead of bidirectional replication from one origin, and so it does not have the conditions necessary to display the leading/lagging strand skew structure that is observed in some bacteria. However, little is known about the replication in other plastid lineages, such as chloroplasts of green algae and the rhodoplasts of red algae. Individual studies have reported skew structures in the chloroplast genome of *Euglena gracilis* [10], which is the product of a secondary endosymbiosis [11] and is known to have a single origin of replication [12], and in the chloroplast of the green alga *Stigeoclonium helveticum* [13], but nothing is known about the distribution of this composition feature across the wide range of plastid genomes that have been sequenced.

Numerous studies have now shown that mutation dynamics in the plant chloroplast genome are complex and provide an excellent model for understanding features that influence mutations. Rates of different mutation types vary dramatically across sites as a function of the surrounding hexanucleotide sequence [14] which has profound implications for sequence analyses such as those that attempt to infer a role for selection on features such as codon usage [15]. If we are to fully understand mutation dynamics in plastid genomes outside of the plants it is critical that we know the distribution of genome skew patterns. Any attempt to analyze plastid sequence data as more becomes available from non-plant lineages will need to account for asymmetry, both in analyses of substitution data and in inferring patterns of selection [10].

In this study, we survey all non-embryophyte plastid genomes for evidence of strand asymmetry centered on a single location. We generate genome walks of the AT and GC base pairs independently [5] and display them as a single 2D walk, with each of the two walks being equivalent to a skew plot with a window size of 1. We then use random walk probability theory to identify which genomes deviate significantly from strand symmetry and compare the locations for the AT and GC walks that give rise to the greatest deviations from the origin. These two locations need not be close together on the genome since they are independent walks, but this is expected to be true in the case of a skew structure. We apply this approach to 311 plastid genomes, excluding seed plants and diatoms, and find evidence for a potential skew structure in several dozen genomes from a wide range of lineages. Although the data are not definitive evidence for a skew structure around a single origin of replication, they provide a basis for further investigation of possible mutation asymmetry and genome structure and other analyses of plastid genome evolution.

## 2. Materials and Methods

Complete genome RefSeq plastid sequences were downloaded from NCBI (https://www.ncbi.nlm.nih.gov/genome/organelle/ accessed on 25 May 2020). Intergenic sequences were extracted using the annotation within each GenBank file. Strand asymmetry was assessed using a random walk and visualized both with a standard skew plot and the random walk. Both whole genome and intergenic sequences were analyzed for each species. Based on the NCBI annotation the genomes were divided into 8 major groups: alveolates, chlorophytes, glaucocystophytes, euglenozoa, haptophytes, rhodophytes, stramenopiles, and streptophytes. There were 5 alveolate taxa in at least 3 different families (in some cases the annotation was uncertain and so complete taxon designation could not be obtained), 118 chlorophytes in at least 34 different families, 3 glaucocystophytes, all in the Cyanophora genus, 17 euglenozoa in 2 different families, 6 haptophytes in at least 3 different families, 72 rhodophytes in at least 37 different families, 75 stramenopiles in at least 37 different families, and 15 streptophytes in 8 different families, for a total of 311 genomes.

Each genome was analyzed as two separate random walks, one for the AT base pairs and one for the GC base pairs [5]. Due to complementarity only one strand needs to be analyzed and in all genomes the strand analyzed was the one provided in the NCBI file. Each walk starts at 0; purines (A or G) are considered, arbitrarily, a step of +1, and pyrimidines (T or C) a step of −1. As the walk proceeds through each nucleotide in the sequence either the AT or GC walk is incremented depending on the base pair at that site. Since we are interested in A-T skew and G-C skew separately, the A+T and G+C subsequences are independent. The null hypothesis under strand symmetry is that in a walk along a single strand, it is equally probable that any AT base pair in the double-stranded sequence will have an A or T on the strand being analyzed. The same holds got G and C. So, in the case of AT subsequence, this means that the AT base pairs are expected to be a random walk with the probability *a* = 0.5 of an A for each step (i.e. equal probability of A or T on one strand). Therefore, *a* = *b* at each step meaning that *a* = 0.5 for an A and *b* = 0.5 for a T at each AT base pair. The two independent 1D walks can then be plotted together in two dimensions.

In a random walk of n steps when *a* = *b* at each step, the probability that the walk will, at some point, deviate at least k steps in one direction from the origin of 0, is given in Equation (1) [16]. Here, n is the length of the walk, or the number of either A + T bases or G + C bases, and S(x) represents the standard normal distribution of the variable x. Significant positive deviation from 0 is a rejection of the null hypothesis that *a* = *b* at each step, providing evidence for a skew towards A (or G), while significant negative deviation is evidence for a skew towards T (or C).

(1) $$p=1-\frac{S\left(k-0.5\right)}{\surd n}$$

**Figure 1:**
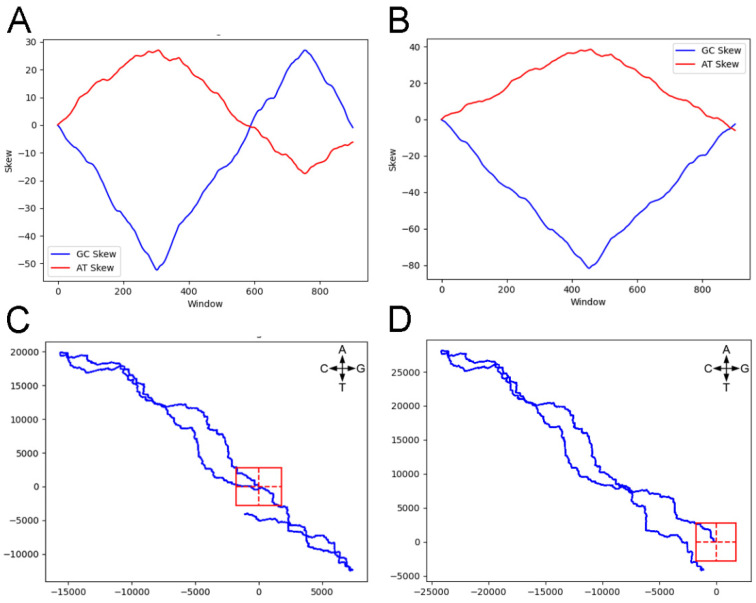
Skew analyses (**A**,**B**) and genome walks (**C**,**D**) for *B. burgdorferi*. The GC skews are shown in blue, while the AT skews are in red. Both use a window size of 10,000 with an overlap of 1000. For the genome walks, the direction of change for each base is indicated; the AT walk is on the vertical axis and the GC walk along the horizontal axis. The origin of the walk is the intersection of the dotted lines and the degree of significant deviation for *p* = 0.01 (see Equation (1)) is outlined by the red box. The GC walk is along the horizontal. The left two plots (**A**,**C**) start at a random genome location, while (**B**,**D**) are “adjusted” to start at the genome location where the AT walk shows maximum deviation from the origin.

Since a genome sequence file can start at any site within the genome, not necessarily the (or an) origin of replication, we find the genome location from which we observe maximum deviation in the AT walk (I_AT_) and the genome location from which we observe maximum deviation in the GC walk (I_GC_) and start the walks from those points. This is equivalent to locating the potential replication origin using the locations from which there is maximum and minimum skew [4]. Figure 1 illustrates this for the strongly-skewed bacterium *Borrelia burgdorferi* B31 (NCBI Accession NC_001318) using both a standard skew plot and a genome walk [5]: this genome sequence file is annotated starting at the origin of replication. Since purines are assigned the +1 step, a walk that tends upwards and to the right is skewed towards A and G, while a walk that tends upwards and to the left is skewed towards T and C. The skew plot was generated using a sliding window of 10,000 nucleotides with an overlap of 1000. For each window, both AT skew ([A − T]/[A + T]) and GC skew ([G − C]/[G + C]) were calculated and added to the cumulative values [2].

The two 1D walks of intergenic regions for each genome were used to determine if there was evidence for a structure consistent with strand asymmetry centered on an origin of replication. Using the intergenic sequences, I_AT_ and I_GC_ were located and it was determined if the deviations of the walks from I_AT_ and I_GC_ were significant using Equation (1) for a sample-corrected *p* = 0.01/(2 × N) where N is the number or genomes analyzed (each with two 1D walks). A genome with an asymmetry structure that is due to replication from a single origin should also meet the criterion that I_AT_ and I_GC_ are located near each other, defined here as I_AT_–I_GC_ being less than 5% of the genome length. These criteria cannot be taken as definitive evidence of skew arising from asymmetric substitution and a single origin of replication, but they provide a basis for examining the potential distribution of skew structure across taxa. All calculations and plots were performed using Python script written by the authors.

## 3. Results

The number of genomes within each of the 8 major groups that show a significant deviation in one or both of the AT and GC walks from I_AT_ and I_GC_ is summarized in Table 1. Deviation from strand symmetry is prevalent in the euglenozoa, and a third of the Rhodophyta in this study show evidence for skew of both AT and GC base pairs. In the genomes where only one of the two walks is significant, it is almost always the AT walk. Of the 32 Chlorophyta with only one significant walk, 25 are the AT walk; of the 35 in Rhodophyta, it is 32 that are AT; and in the stramenopiles 8 of the 9 have a significant AT walk. In those genomes for which both walks are significant, all of them show a skew towards G and T on the same strand with the exception of the three chlorophytes, which show a skew towards G and A on the same strand.

**Figure 2:**
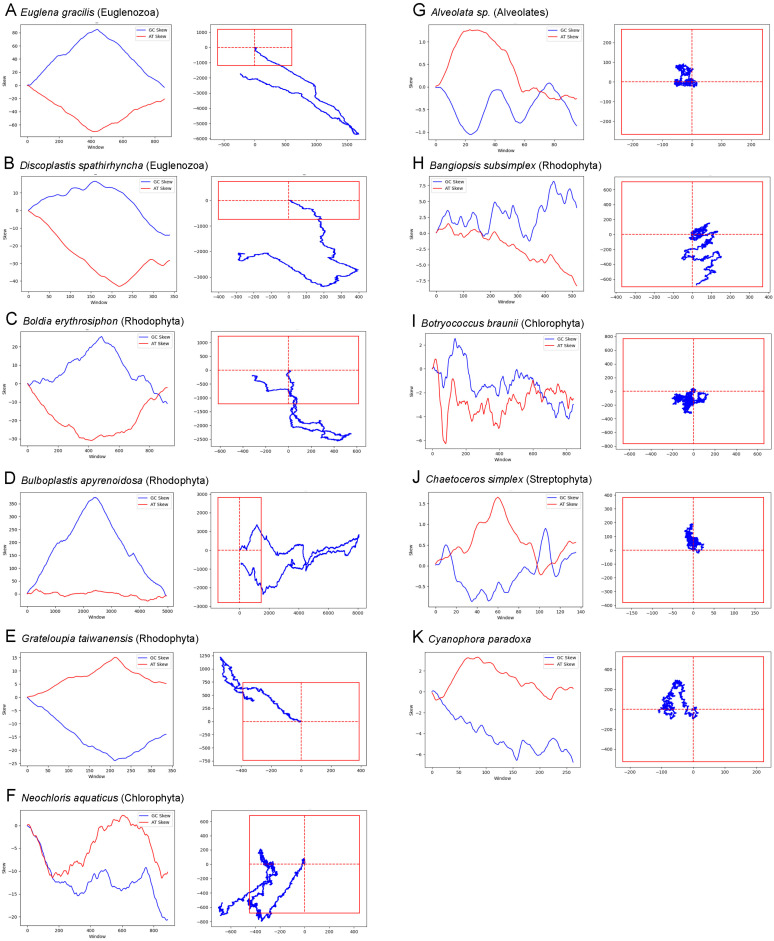
Skew plots and walks for 6 representative genomes with evidence for a skew pattern (**A–F**) and 5 that do not (**G**–**K**). The skew was calculated using a window size of 1000 and an overlap of 100. For the genome walks, the direction of change for each base is indicated; the AT walk is on the vertical axis and the GC walk along the horizontal axis. The origin of each walk is the intersection of the dashed red lines and the outer box represents the significance point as described in Section 2.

Table 2 shows the number of genomes that have significant AT and/or GC walks along with co-location of IAT and IGC as defined in Section 2. Figure 2 shows the walk and intergenic skew diagrams from representative genomes that meet the criteria of at least one significant skew and co-location, contrasted with representative genomes in which neither walk was significant.

**Figure 3:**
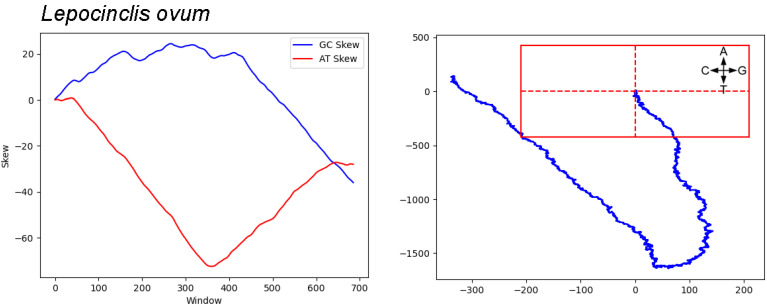
Cumulative skews and genome walk of intergenic regions from the euglenozoa *Lepocinclis ovum*. The skew was calculated using a window size of 1000 and an overlap of 100. The origin of the walk is the intersection of the dashed red lines and the outer box represents the significance point as described in Section 2. For the genome walk, the direction of change for each base is indicated; the AT walk is on the vertical axis and the GC walk along the horizontal axis.

The three euglenozoa that meet the criteria of co-location of I_AT_ and I_GC_ but have only one significant walk (see Table 2) are the three members of the genus Lepocinclis. The skew and walk results for intergenic regions of *Lepocinclis ovum* are shown in Figure 3. Curiously, unlike *Euglena gracilis* (see Figure 2), the GC skew in this species appears to be much more pronounced in one half of the genome.

**Figure 4:**
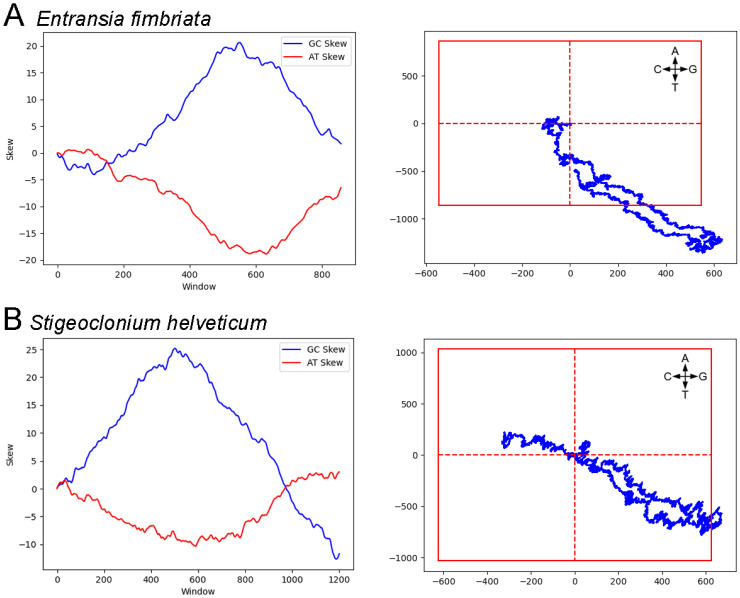
Cumulative skews and genome walk for the intergenic regions of the streptophyte *Entransia fimbriata* (**A**) and the chlorophyte *Stigeoclonium helveticum* (**B**). The skew was calculated using a window size of 1000 and an overlap of 100. For the genome walks, the direction of change for each base is indicated; the AT walk is on the vertical axis and the GC walk along the horizontal axis. The origin of each walk is the intersection of the dashed red lines and the outer box represents the significance point as described in the Section 2.

The genomes that show significant evidence for a skew pattern are widely distributed across lineages, regardless of whether we consider skew alone, or significant skew of at least one walk along with the co-location of I_AT_ and I_GC_. Within the 13 Chlorophyta meeting the latter criteria, there are representatives of the three major orders, Chlorophyceae, Trebouxiophyceae, and Ulvophyceae, and within the 28 rhodophyte genomes there are members of the orders Florideophyceae, Bangiophyceae, and Compsopogonophyceae. Of the five genomes within the Stramenopiles meeting these criteria, there are two members of the phylum Ochrophyta, each from a different order, and three members of the Bacillariophyta, each from a different family. The one streptophyte in this category is *Entransia fimbriata* (Figure 4), a member of the Zygnemataceae, although there are three other members of this family in the study that do not show evidence, by our criteria, of a skew pattern. The walk in *E. fimbriata* is just barely significant meaning that the skew is relatively weak, and it is possible that this could be a remnant of an ancestral bias or false positive. In general, there is little evidence for a skew pattern within the Streptophytes.

If we look at the degree of skew, measured by the amount of deviation from the walk origin, the genomes with the strongest skew are predominantly euglenozoas and rhodophytes. Of the 25 genomes with the lowest probability deviations (based on Equation (1)) for the AT walk, 12 are rhodophytes and 13 are euglenozoas, and of the 25 genomes with the lowest probabilities for the GC walk, 15 are euglenozoas, 9 are rhodophytes and 1 is a chlorophyte.

**Figure 5:**
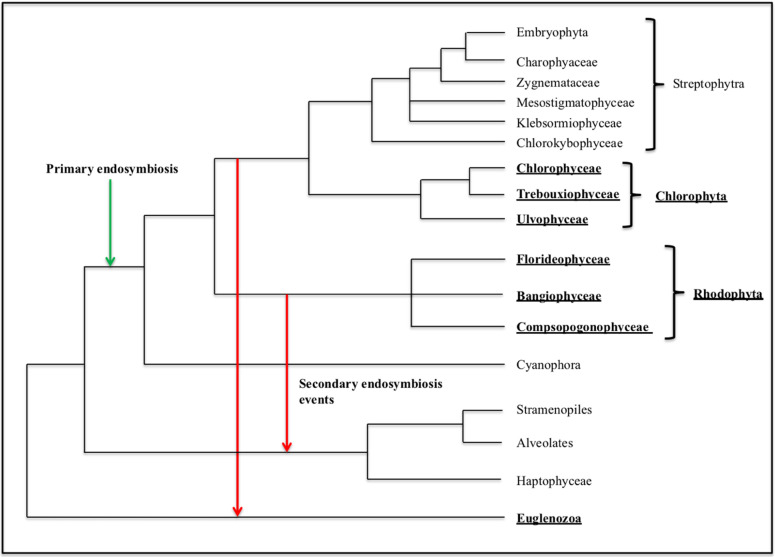
Distribution of skew pattern genomes in a phylogenetic framework using the general relationships in ref. [17]. The lineages with multiple skew-pattern genomes, here defined as those that have at least one significant walk and co-location of I_AT_ and I_GC_, are in bold and underlined.

Of note, the chlorophyte *Stigeoclonium helveticum*, which had previously been noted to have a skew structure [13], has a significant GC walk, although the AT walk is not significant (Figure 4). Although I_AT_ and I_GC_ are 7.3% of the genome apart, it is likely that this genome has a skew pattern since co-location is of less importance when only one walk is significant. The phylogenetic distribution of genomes with evidence for a skew pattern is summarized in Figure 5.

## 4. Discussion

The data provide evidence for a skew pattern in plastid genomes from a wide range of taxa outside of the land plants, which were excluded given their mode of replication [9]. By skew pattern we refer to a specific pattern of asymmetry that is consistent with replication from a single origin combined with strand-biased substitution, a genome composition structure that is fairly common in eubacteria [1,3,4]. In these bacterial genomes, the skew pattern means that there is a compositional difference between leading and lagging strands, a difference that results from a combination of selection, including a coding strand bias, and strand-biased mutation dynamics [1,4]. Since the genome plots shown here are limited to the intergenic sequences, it is likely that skew in these plastid genomes is predominantly due to a bias in mutation dynamics. This would include differences in the repair process, which can be considered an extension of mutations from an evolutionary perspective. Thus, the strand bias could partly result from differences in repair between the leading and lagging strands, which could arise from the vastly different time spent in the single stranded state by the two strands during replication. However, since little is known about plastid genome replication and repair [8] it is difficult to know what factors could contribute to asymmetry. 

Although the genome walk data are consistent with a skew pattern in a number of lineages, whether or not the asymmetries detected are due to a strand bias centered on a single origin of replication cannot be determined from composition alone. What needs to be established is the location(s) of replication origins so that these can be compared to the I_AT_ and I_GC_ regions identified here. Despite this limitation, the similarity to bacteria is strongly suggestive. Additionally, the observation of a particularly strong pattern in the euglenozoa, along with the fact that there is a single replication origin in *E. gracilis* [12], supports the explanation that strand-biased mutation is responsible for the skew pattern in this group. At the very least, the apparent relationship between skew and replication in *E. gracilis* indicates that such a pattern is possible in plastid genomes and that it could have existed in the original endosymbiont.

Another interesting feature of the data is the consistent skew towards G and T on one strand (A and C on the complement), with the exception of the chlorophytes, which show a skew towards purines on the same strand. The existence of a common pattern is not what we would expect if the skew patterns in different genomes were a result of different causes, or false positives. Moreover, the difference between chlorophytes and the other lineages would require at least one evolutionary shift in the underlying mutation pattern. If replication and leading/lagging strand asymmetry is the cause of the skew pattern then it would be intriguing to understand how such a shift in asymmetry could occur. Since it would seem to require a significant change in core processes of repair and/or replication error, it may be worthwhile examining the repair pathways in euglenozoan and chlorophytes. Given the data here we might predict that there is a significant difference in the repair pathways utilized by the chloroplasts in these lineages. Why such a shift would occur is also an intriguing question: does it represent a selective shift in repair mechanisms or a random change in which processes are active? Since both the GT and AG strand biases are also observed in eubacteria [4], it would be interesting to understand the dynamics of such an evolutionary shift.

Two points, when considered together, suggest that the skew pattern is an ancestral characteristic of the original endosymbiotic bacterium. One point is the strong skew pattern observed in some lineages, particularly the euglenozoa, and the similarity of this pattern to a common eubacterial genome feature. As noted above, this provides strong evidence that the observations are due in part to a replication-associated skew pattern in plastid genomes. The second point is the widespread distribution of the skew pattern across plastid lineages, which would require several independent evolutionary appearances of the conditions responsible for the skew pattern if this were a derived feature. We propose that the more likely scenario is that the skew pattern is ancestral, with multiple evolutionary loses due to a change in replication mechanism and/or changes in mutation properties. Under this model it is also possible that the asymmetry in some genomes is the result of ancestral, not current, mutational skew or selection, and the strand asymmetry is now decaying.

Altogether, it seems likely that bidirectional replication with strand bias plays a role in generating this genome composition structure, but more direct experimental data will be needed to determine the underlying cause(s) of the skew pattern observed. What needs to be explored are the differences in the mode of replication across these lineages so that we can determine whether any differences in skew are due to differences in replication as opposed to differences in mutation dynamics. This could provide valuable insights into the evolution of genome replication and the evolution of mutation properties. Regardless of causal mechanism, though, the existence of strong skew in some plastid lineages raises some interesting points for evolutionary analyses. Amongst these would be phylogenetic analyses that assume uniform substitution models across genes, and analyses of genome composition patterns, such as codon usage. A full understanding of codon usage bias, and the relative contributions of selection and mutation to plastid codon bias [17] requires an adjustment for genome location in genomes with a skew pattern. Genome location will also be important if we wish to extend recent analyses from angiosperms that use substitution data to uncover the role of neighboring base influences on mutation dynamics [14] to non-plant lineages, and in studies that compare intergenic and coding composition to assess selection [15]. Overall, what is emerging from this and other recent studies is that the plastid could provide many valuable future insights about genome structure, complex mutation dynamics, and the relationships between these two things.

## Figures and Tables

**Table 1:** Number of genomes showing significant walk deviation when relative location of I_AT_ and I_GC_ is not considered.

		1 Walk Sig.		2 Walks Sig.	
Group	No. Taxa	Number	Percent	Number	Percent
Alveolates	5	1	20.0%	0	-
Chlorophyta	118	35	29.7%	3	2.5%
Cyanophora	3	0	-	0	-
Eugleonozoa	17	17	100%	16	94.1%
Haptophyceae	6	1	16.7%	0	-
Rhodophyta	72	59	81.9%	24	33.3%
Stramenopiles	75	9	12.0%	0	-
Streptophyta	15	2	13.3%	1	6.7%

**Table 2:** Number of genomes showing significant walk deviation when I_AT_–I_GC_ is constrained to less than 5% of the genome length.

		1 Walk Sig.		2 Walks Sig.	
Group	No. Taxa	Number	Percent	Number	Percent
Alveolates	5	1	20.0%	0	-
Chlorophyta	118	13	11.0%	2	1.7%
Cyanophora	3	0	-	0	-
Eugleonozoa	17	14	82.4%	9	52.9%
Haptophyceae	6	1	16.7%	0	-
Rhodophyta	72	28	38.9%	8	11.1%
Stramenopiles	75	5	6.7%	0	-
Streptophyta	15	1	6.7%	1	6.7%

## Data Availability

The genome walk plots of all genomes are available at Dryad (https://doi.org/10.5061/dryad.n2z34tn1c).
